# Towards the conservation of parasitoid wasp species in Canada: Preliminary assessment of Microgastrinae (Hymenoptera: Braconidae)

**DOI:** 10.3897/BDJ.2.e1067

**Published:** 2014-02-27

**Authors:** Jose L Fernandez-Triana

**Affiliations:** † Canadian National Collection of Insects, Ottawa, and Biodiversity Institute of Ontario, University of Guelph, Ottawa, Canada

**Keywords:** Hymenoptera, Braconidae, Microgastrinae, parasitoid wasps, species conservation, species candidate lists, COSEWIC

## Abstract

This paper is the first to consider braconid parasitoid wasps in conservation efforts in Canada. Out of the 28 genera of the subfamily Microgastrinae (Hymenoptera: Braconidae) present in the country, 13 genera were studied and 16 species were identified as potential candidates to be included in the Species Candidate Lists of COSEWIC (The Committee on the Status of Endangered Wildlife in Canada). For every selected species a brief summary of its broad geographical distribution is provided, with detailed and in many cases new information of its distribution and collecting dates in Canada, hosts (Lepidoptera) if known, and color pictures of all wasp species. A preliminary assessment is made using Prioritization Criteria developed by COSEWIC, and some general recommendations are made based in those analyses.

## Introduction

The parasitoid wasps (Hymenoptera) have been considered as a keystone group that has a disproportionately large role in maintaining the diversity of other animals and plants (LaSalle and Gauld 1993). This is mainly a result of the profound and often highly specialized interactions between them and other organisms (particularly plants and other insects), as well as their inherent contribution to biodiversity by being a large and ramified group (Shaw and Hochberg 2001). In spite of its importance, parasitoid wasps have rarely been considered in conservation biology efforts. For example, there are no parasitoid wasps among the 302 species of Hymenoptera included in The IUCN Red List of Threatened Species (http://www.iucnredlist.org/). Very few countries have provided information or strategies to be used in species conservation of parasitoid wasps (e.g. Söderman et al. 2010, Hansen et al. 2010, Ward et al. 2012).

The Committee on the Status of Endangered Wildlife in Canada (COSEWIC, http://www.cosewic.gc.ca/) exists to provide advice regarding the status of wildlife species that are nationally at risk of extinction or extirpation. Its committee of experts assesses and designates which wildlife species are in some danger of disappearing from the national territory. As part of its work, COSEWIC produces a Species Candidate Lists based on Prioritization Criteria (http://www.cosewic.gc.ca/eng/sct3/index_e.cfm#p1). There has never been a species of braconid parasitoid wasp being considered by COSEWIC, mostly because lack of available information about any potential species to be considered.

This paper is an effort to provide new and/or updated information about species of Microgastrinae wasps (Hymenoptera: Braconidae) with potential to be considered by COSEWIC to be included in future Species Candidate Lists. Microgastrinae is one of the most diverse and important groups of parasitoids wasps (Yu et al. 2012, Whitfield 1995, Rodriguez et al. 2013).

## Materials and methods

This paper is mostly based on the study of specimens from the Canadian National Collection of Insects (CNC). In a few cases, examination of photographs and distribution records of specimens deposited in the Biodiversity Institute of Ontario (BIO) was done by accessing public data available in the Barcode of Life Data Systems (http://www.boldsystems.org/).

Out of the 28 genera of Microgastrinae (Hymenoptera: Braconidae) present in Canada (Fernandez-Triana 2010), 13 genera (representing almost 80 species) were examined for species of potential interest in conservation efforts. A total of 16 species were identified as meeting the Prioritization Criteria by COSEWIC (see below for criteria used), and were selected for this paper. Other genera, where more taxonomic work is needed (e.g., *Cotesia*, *Dolichogenidea*, *Microplitis*, *Pholetesor*) will be evaluated in future works. There are 194 described species of Microgastrinae in Canada (Fernandez-Triana 2010, Yu et al. 2012).

For every selected species, a brief summary of its broad geographical distribution is provided, with detailed and in many cases new information of its distribution and collecting dates in Canada, hosts (Lepidoptera) if known, and color pictures of the wasp species. A preliminary assessment is then made using the prioritization criteria developed by COSEWIC (http://www.cosewic.gc.ca/eng/sct0/appdx_e1_2_e.cfm): Proportion of the species global range in Canada, Details on existing global conservation status, Canadian population size and trends, Threats, Small extent of occurrence or area of occupancy are also discussed. That information is provided to comply with COSEWIC standards when considering potential candidate species. Meeting the COSEWIC criteria allows a species to be evaluated and eventually incorporated to the COSEWIC Species Candidate Lists – and once in those lists, the species can be studied further and considered for the IUCN Red List of Threatened Species, if applicable.

Most of the photos were taken with a Keyence VHX-1000 Digital Microscope, using a lens with a range of 13–130 ×. Some of the species were photographed with a Canon EOS 60D with MPE-65 lenses (aperture: 4.0, ISO: 100, CR2 format images), and a 600EX-RT Speedlight (manual) flash; the camera was mounted on a Kaiser copy stand with a Z-stepper (Stackshot) to allow for taking of multiple images. Multiple images through the focal plane were taken of a structure and these were combined to produce a single in-focus image. For the pictures taken with the Canon camera, the Zerene Stacker program (http://zerenesystems.com/cms/stacker) was used; the software associated with the Keyence System produced the focused images taken with that camera.

Maps with the distribution in Canada of all species were generated using SimpleMappr (http://www.simplemappr.net/).

## Taxon treatments

### Alphomelon winniewertzae

Deans, 2003

#### Materials

- **Type status:** Other material. **Occurrence:** recordedBy: Jose Fernandez-Triana; individualCount: one; sex: female; **Location:** country: Canada; stateProvince: Ontario; verbatimLocality: Marmora; **Event:** eventDate: 8.vii.1952; **Record Level:** institutionCode: CNC
- **Type status:** Other material. **Occurrence:** recordedBy: Jose Fernandez-Triana; individualCount: one; sex: female; **Location:** country: Canada; stateProvince: Quebec; verbatimLocality: Old Chelsea, Gatineau Park, Summit of King Mountain; verbatimElevation: 350 m; **Event:** eventDate: 11.viii.1965; **Record Level:** collectionID: CNC

#### Distribution

Figs 1, 2

This genus is distributed from the Neotropics (Costa Rica, Mexico) to central and eastern United States (Yu et al. 2012). Deans et al. 2003 mentioned *Alphomelon winniewertzae* from Canada (Ontario, Marmora, one female specimen deposited in the CNC), and Fernandez-Triana 2010 recorded the species as also present in the province of Quebec, without giving more details. Here complete information of that second record is provided for the first time (Quebec, Old Chelsea, Gatineau Park, Summit of King Mountain, one female specimen deposited in the CNC). The species has been reported by Deans et al. 2003 as a parasitoid of *Calpodes ethlius* and *Euphyes vestris* (Lepidoptera: Hesperidae). Based on the information available, *Alphomelon winniewertzae* could be distributed in Canada in an area between the rivers Ottawa and Saint Lawrence (44–45°N, 77–78°W). That represents less than 5% of the global range of the species. *Alphomelon* is mostly a Neotropical genus, with only three species reaching the Nearctic (mostly southern and eastern US), and *Alphomelon winniewertzae* is the only known in Canada and the northenmost species of the genus.

#### Conservation

**Assessment using the prioritization criteria developed by COSEWIC.** Existing global conservation status: None (species is not listed on Natureserve nor has it been assigned a Canadian national conservation status rank). Canadian population size and trends: No information on population size is available. Threats: Residential and commercial development – high (most of the areas where the species occur in Canada are already heavily populated); Agriculture and aquaculture – unknown; Human intrusions and disturbance – medium; Natural system modifications – high (alteration of the natural areas currently protected would likely extirpate the species from Canada); Invasive and other problematic species and genes – unknown but likely low, unless another wasp species parasitizing the same host would be introduced (and then competing for the same host, an scenario not likely to occur); Climate change and severe weather – unknown but likely low (climate change increasing the temperatures would not affect much the presence of this species in Canada, because it is already distributed in warmer areas). Small extent of occurrence or area of occupancy: Recorded from two localities in Canada. Limiting biological factors: Unknown.

### Apanteles samarshalli

Fernández-Triana, 2010

#### Materials

- **Type status:** Paratype. **Occurrence:** recordedBy: Jose Fernandez-Triana; individualCount: 1; sex: female; **Location:** country: Canada; stateProvince: Ontario; verbatimLocality: Rondeau Provincial Park; **Event:** samplingProtocol: Malaise trap; eventDate: 19.viii-11.ix.1973; **Record Level:** institutionCode: CNC
- **Type status:** Other material. **Occurrence:** individualCount: 1; sex: female; **Location:** country: Canada; stateProvince: Ontario; verbatimLocality: Point Pelee National Park, Cactus Field, Cedar/Savannah; verbatimElevation: 168 m; verbatimLatitude: 41.939; verbatimLongitude: -82.516; **Event:** eventDate: 5-12.ix.2012; **Record Level:** collectionID: BIOUG03931-F03; institutionCode: BIO
- **Type status:** Other material. **Occurrence:** individualCount: 1; sex: female; **Location:** country: Canada; stateProvince: Ontario; verbatimLocality: near Brockville; verbatimElevation: 112 m; verbatimLatitude: 44.621; verbatimLongitude: -75.773; **Event:** eventDate: 10.vi.2010; **Record Level:** collectionID: BIOUG01035-G03; institutionCode: BIO
- **Type status:** Other material. **Occurrence:** individualCount: 2; sex: female; **Location:** country: Canada; stateProvince: Ontario; verbatimLocality: near Brockville; verbatimElevation: 112 m; verbatimLatitude: 44.621; verbatimLongitude: -75.773; **Event:** eventDate: 28.vii.2010; **Record Level:** collectionID: BIOUG01035-G03; institutionCode: BIO
- **Type status:** Other material. **Occurrence:** individualCount: 1; sex: female; **Location:** country: Canada; stateProvince: Ontario; verbatimLocality: near Brockville; verbatimElevation: 112 m; verbatimLatitude: 44.621; verbatimLongitude: -75.773; **Event:** eventDate: 18.vi.2010; **Record Level:** collectionID: BIOUG01035-G03; institutionCode: BIO
- **Type status:** Other material. **Occurrence:** individualCount: 1; sex: female; **Location:** country: Canada; stateProvince: Ontario; verbatimLocality: near Brockville; verbatimElevation: 112 m; verbatimLatitude: 44.621; verbatimLongitude: -75.773; **Event:** eventDate: 3.viii.2010; **Record Level:** collectionID: BIOUG01035-G03; institutionCode: BIO
- **Type status:** Other material. **Occurrence:** individualCount: 2; sex: female; **Location:** country: Canada; stateProvince: Ontario; verbatimLocality: near Brockville; verbatimElevation: 112 m; verbatimLatitude: 44.621; verbatimLongitude: -75.773; **Event:** eventDate: 5.viii.2010; **Record Level:** collectionID: BIOUG01035-G03; institutionCode: BIO

#### Distribution

Figs 3, 4

The distribution of this species was originally reported from southern Canada (Rondeau Provincial Park) to the Florida keys in the United States (Fernandez-Triana 2010). Subsequent work has expanded the known range towards tropical areas such as Mexico (Fernández-Flores et al. 2013) and Costa Rica (Fernandez-Triana et al. 2014). Canada remains as the northern limit, representing less than 5% of the global range of the species. Until now only a single female specimen, collected in 1973 in Rondeau Provincial Park, was known from Canada (Fernandez-Triana 2010); here additional specimens are reported from two new localities (Ontario, Point Pelee National Park and Brockville, specimens deposited in BIO). The new records, all from 2012, confirm the current presence of *Apanteles samarshalli* in Canada, significantly expand the distribution of the species in southern Ontario, and slightly expand its northern range up to 45°N. Nothing is known about the hosts caterpillars parasitized by *Apanteles samarshalli*, but most of the specimens have been collected in hammock forests, and most of the known localities share in common the presence of oaks trees (genus *Quercus*) or cedar (*Juniperus*, *Cupressus*).

#### Conservation

**Assessment using the prioritization criteria developed by COSEWIC.** Existing global conservation status: None (species is not listed on Natureserve nor has it been assigned a Canadian national conservation status rank). Canadian population size and trends: No information on population size is available. Threats: Residential and commercial development – medium to high (some of the areas where the species occur in Canada are already heavily populated); Agriculture and aquaculture – unknown; Human intrusions and disturbance – medium; Natural system modifications – high (alteration of the natural areas currently protected would likely extirpate the species from Canada); Invasive and other problematic species and genes – unknown but likely low, unless another wasp species parasitizing the same host would be introduced (and then competing for the same host, an scenario not likely to occur); Climate change and severe weather – unknown but likely low (climate change increasing the temperatures would not affect much the presence of this species in Canada, because it is already distributed in warmer areas). Small extent of occurrence or area of occupancy: Recorded from a few localities in southern Ontario. Limiting biological factors: Unknown.

### Clarkinella canadensis

Mason, 1981

#### Materials

- **Type status:** Holotype. **Occurrence:** recordedBy: Jose Fernandez-Triana; individualCount: 1; sex: female; **Location:** country: Canada; stateProvince: Ontario; verbatimLocality: Ottawa; **Event:** eventDate: 28.vii.1959; **Record Level:** institutionCode: CNC
- **Type status:** Other material. **Occurrence:** recordedBy: Jose Fernandez-Triana; individualCount: 1; sex: female; **Location:** country: Canada; stateProvince: Ontario; verbatimLocality: Ottawa; **Event:** eventDate: 30.vii.2007; **Record Level:** institutionCode: CNC
- **Type status:** Other material. **Occurrence:** recordedBy: Jose Fernandez-Triana; individualCount: 1; sex: female; **Location:** country: Canada; stateProvince: Ontario; verbatimLocality: Ottawa; **Event:** eventDate: 8.iX.2007; **Record Level:** institutionCode: CNC

#### Distribution

Figs 5, 6

This species was described from a single female from Canada (Ontario, Ottawa, holotype deposited in the CNC). Fernandez-Triana 2010 mentioned two additional specimens from the same locality, without giving more details. Here complete information of those records is provided for the first time. The new data confirms the current presence of *Clarkinella canadensis* in Canada, so far only known from a single locality (100% of the global range of the species). *Clarkinella* is mostly a Neotropical genus, with only *Clarkinella canadensis* reaching the Nearctic, and no more species expected from North America (Fernandez-Triana 2010, Whitfield 1995). Nothing is known about the hosts caterpillars parasitized by this braconid wasp.

#### Conservation

**Assessment using the prioritization criteria developed by COSEWIC.** Existing global conservation status: None (species is not listed on Natureserve nor has it been assigned a Canadian national conservation status rank). Canadian population size and trends: No information on population size is available, but the only known specimens are all from a single locality, and have been repeatedly collected over a span of 50 years, usually during late July (but with one record from early September). Threats: Residential and commercial development – high (the single area where the species occurs in Canada is already heavily populated); Agriculture and aquaculture – unknown; Human intrusions and disturbance – medium; Natural system modifications – high (alteration of the area would likely extirpate the species from Canada); Invasive and other problematic species and genes – unknown but likely low, unless another wasp species parasitizing the same host would be introduced (and then competing for the same host, an scenario not likely to occur); Climate change and severe weather – unknown. Small extent of occurrence or area of occupancy: Recorded from one locality in Canada (the only locality known for the species). Limiting biological factors: Unknown.

### Deuterixys pacifica

Whitfield, 1985

#### Materials

- **Type status:** Other material. **Occurrence:** recordedBy: Jose Fernandez-Triana; individualCount: 1; sex: female; **Location:** country: Canada; stateProvince: British Columbia; verbatimLocality: Robson; **Event:** eventDate: 13.v.1947; **Record Level:** institutionCode: CNC

#### Distribution

Figs 7, 8

This species is rather widely distributed in western North America from Mexico to British Columbia, with most of the records from California, United States (Whitfield and Oltra-Moscardo 2004, Whitfield 1985). It is only know in Canada from one female specimen (British Columbia, specimen deposited in the CNC), by far the northernmost record, and representing less than 5% of the global range of the species. It has been reared from two species of *Bucculatrix* (Lepidoptera: Bucculatrigidae) feeding on plants of *Artemisia* spp., *Baccharis pilularis*, and *Iva axillaris* (information summarized in Yu et al. 2012).

#### Conservation

**Assessment using the prioritization criteria developed by COSEWIC.** Existing global conservation status: None (species is not listed on Natureserve nor has it been assigned a Canadian national conservation status rank). Canadian population size and trends: No information on population size is available. Threats: Residential and commercial development – high (the single area where the species occur in Canada is populated); Agriculture and aquaculture – unknown; Human intrusions and disturbance – medium; Natural system modifications – high (alteration of the area would likely extirpate the species from Canada); Invasive and other problematic species and genes – unknown but likely low, unless another wasp species parasitizing the same host would be introduced (and then competing for the same host, an scenario not likely to occur); Climate change and severe weather – unknown but likely low (climate change increasing the temperatures would not affect much the presence of this species in Canada, because it is already distributed in warmer areas). Small extent of occurrence or area of occupancy: Recorded from one locality in Canada. Limiting biological factors: Probably none.

### Diolcogaster garmani

(Ashmead, 1900)

#### Materials

- **Type status:** Other material. **Occurrence:** recordedBy: Jose Fernandez-Triana; individualCount: 1; sex: female; **Location:** country: Canada; stateProvince: Ontario; verbatimLocality: Thamesville; **Event:** eventDate: 20.vi.1962; **Record Level:** institutionCode: CNC

#### Distribution

Figs 9, 10

This species is distributed in central and eastern United States (Yu et al. 2012). Fernandez-Triana 2010 recorded the species as also present in the province of Ontario, without giving more details. Here complete information of that specimen is provided for the first time (Ontario, Thamesville, one female deposited in the CNC). This represent the northernmost record and less than 5% of the global range of the species. *Diolcogaster garmani* has been recorded as a parasitoid of *Ogdoconta cinereola* (Lepidoptera: Noctuidae) in the United States (information summarized in Yu et al. 2012).

#### Conservation

**Assessment using the prioritization criteria developed by COSEWIC.** Existing global conservation status: None (species is not listed on Natureserve nor has it been assigned a Canadian national conservation status rank). Canadian population size and trends: No information on population size is available. Threats: Residential and commercial development – high (the single area where the species occurs in Canada is already heavily populated); Agriculture and aquaculture – unknown; Human intrusions and disturbance – medium; Natural system modifications – high (alteration of the area would likely extirpate the species from Canada); Invasive and other problematic species and genes – unknown but likely low, unless another wasp species parasitizing the same host would be introduced (and then competing for the same host, an scenario not likely to occur); Climate change and severe weather – unknown but likely low (climate change increasing the temperatures would not affect much the presence of this species in Canada, because it is already distributed in warmer areas). Small extent of occurrence or area of occupancy: Recorded from one locality in Canada. Limiting biological factors: Host distribution (limited to southern Ontario and Quebec) may affect the distribution of the wasp in Canada.

### Distatrix carolinae

Fernández-Triana, 2010

#### Materials

- **Type status:** Holotype. **Occurrence:** recordedBy: Jose Fernandez-Triana; individualCount: 1; sex: female; **Location:** country: Canada; stateProvince: Quebec; verbatimLocality: Old Chelsea, Gatineau Park, Summit of King Mountain; verbatimLatitude: 45°29'16" N; verbatimLongitude: 75°51'52" W; **Event:** eventDate: 26.vi.1977; **Record Level:** institutionCode: CNC

#### Distribution

Figs 11, 12

This species was described from a single female from Canada (Quebec, Gatinaeu Park, Old Chelsea, Summit of King Mountain, holotype deposited in the CNC) (Fernandez-Triana 2010). So far this is the only known locality of *Distatrix carolinae* (100% of the global range of the species), and it also represents the northernmost record of the genus *Distatrix*.

#### Conservation

**Assessment using the prioritization criteria developed by COSEWIC.** Existing global conservation status: None (species is not listed on Natureserve nor has it been assigned a Canadian national conservation status rank). Canadian population size and trends: No information on population size is available. Threats: Residential and commercial development – low (the single area where the species occurs in Canada has some degree of protection); Agriculture and aquaculture – low; Human intrusions and disturbance – high (the park where the species occurs has a relative heavy load of visitors); Natural system modifications – high (alteration of the natural area currently protected would likely extirpate the species from Canada); Invasive and other problematic species and genes – unknown but likely medium; Climate change and severe weather – unknown but likely to be high. Small extent of occurrence or area of occupancy: Recorded from one locality in Canada (the only locality known for the species). Limiting biological factors: Unknown.

### Exix columbica

Mason, 1981

#### Materials

- **Type status:** Holotype. **Occurrence:** recordedBy: Jose Fernandez-Triana; individualCount: 1; sex: female; **Location:** country: Canada; stateProvince: British Columbia; verbatimLocality: Verde Creek, northeast from Copper Mountain; **Event:** eventDate: 21.vii.1949; **Record Level:** institutionCode: CNC

#### Distribution

Figs 13, 14

This species was described from a single female from Canada (British Columbia, Verde Creek, northeast from Copper Mountain, holotype deposited in the CNC). *Exix* is mostly a Neotropical genus, with only *Exix columbica* reaching the Nearctic, and no more species expected from North America (Fernandez-Triana 2010, Mason 1981, Whitfield 1995). Nothing is known about the hosts caterpillars parasitized by this braconid wasp.

#### Conservation

**Assessment using the prioritization criteria developed by COSEWIC.** Existing global conservation status: None (species is not listed on Natureserve nor has it been assigned a Canadian national conservation status rank). Canadian population size and trends: No information on population size is available. Threats: Residential and commercial development – medium to high (the single area where the species occurs is populated); Agriculture and aquaculture – unknown; Human intrusions and disturbance – medium; Natural system modifications – high (alteration of the area would likely extirpate the species from Canada); Invasive and other problematic species and genes – unknown but likely low, unless another wasp species parasitizing the same host would be introduced (and then competing for the same host, an scenario not likely to occur); Climate change and severe weather – unknown. Small extent of occurrence or area of occupancy: Recorded from one locality in Canada (the only locality known for the species). Limiting biological factors: Unknown.

### Lathrapanteles heleios

Williams, 1985

#### Materials

- **Type status:** Other material. **Occurrence:** recordedBy: Jose Fernandez-Triana; individualCount: 2; **Location:** country: Canada; stateProvince: Ontario; verbatimLocality: Aylmer West; **Event:** eventDate: 24-31.viii.1972; **Record Level:** institutionCode: CNC
- **Type status:** Other material. **Occurrence:** recordedBy: Jose Fernandez-Triana; individualCount: 2; **Location:** country: Canada; stateProvince: Ontario; verbatimLocality: Leeds-Grenville County forest; verbatimLatitude: 44.6288; verbatimLongitude: -76.359; **Event:** eventDate: 1.x.2008; **Record Level:** institutionCode: CNC
- **Type status:** Other material. **Occurrence:** recordedBy: Jose Fernandez-Triana; individualCount: 2; **Location:** country: Canada; stateProvince: Ontario; verbatimLocality: Mer Blue, Ottawa; **Event:** eventDate: 10.vi.1975; **Record Level:** institutionCode: CNC
- **Type status:** Other material. **Occurrence:** recordedBy: Jose Fernandez-Triana; individualCount: 5; **Location:** country: Canada; stateProvince: Ontario; verbatimLocality: Ottawa, city garden; verbatimLatitude: 45.3561; verbatimLongitude: -75.707; **Event:** eventDate: 1.ix.2007; **Record Level:** institutionCode: CNC
- **Type status:** Other material. **Occurrence:** recordedBy: Jose Fernandez-Triana; individualCount: 1; **Location:** country: Canada; stateProvince: Ontario; verbatimLocality: Spencerville; **Event:** eventDate: 15.viii.1978; **Record Level:** institutionCode: CNC

#### Distribution

Figs 15, 16

Since the original description (Williams 1985) this species is known to be distributed in southern Ontario (Ontario, Mer Blue, and Spencerville). Here additional specimens recently collected are reported from two new localities in the same region (Ottawa city garden, and Leeds-Grenville County forest). Altogether, 43 specimens are deposited in the CNC, comprising 100% of the global range of the species. The genus *Lathrapanteles* has three described species in the Nearctic and one in the Neotropics, but *Lathrapanteles heleios* is the only Canadian endemic, and the species with the most restricted distribution within the genus. Nothing is known about the hosts caterpillars parasitized by this braconid wasp.

#### Conservation

**Assessment using the prioritization criteria developed by COSEWIC.** Existing global conservation status: None (species is not listed on Natureserve nor has it been assigned a Canadian national conservation status rank). Canadian population size and trends: No information on population size is available, but the species has been collected over a span of 33 years, usually on early July (but ranging from June to early October). Threats: Residential and commercial development – medium to high (some of the areas where the species occur in Canada are already heavily populated); Agriculture and aquaculture – unknown; Human intrusions and disturbance – medium; Natural system modifications – high (alteration of the natural areas currently protected would likely extirpate the species from Canada); Invasive and other problematic species and genes – unknown but likely low, unless another wasp species parasitizing the same host would be introduced (and then competing for the same host, an scenario not likely to occur); Climate change and severe weather – unknown. Small extent of occurrence or area of occupancy: Recorded from a few localities in southern Ontario. Limiting biological factors: Unknown.

### Microgaster deductor

Nixon, 1968

#### Distribution

Figs 17, 18

The distribution and other data about this species was revised and updated recently by Fernandez-Triana 2014. It is of interest because some partial evidence suggests that the species might be shifting towards northern localities, although more study is necessary. Nothing is known about the hosts caterpillars parasitized by this braconid wasp.

#### Conservation

**Assessment using the prioritization criteria developed by COSEWIC.** Existing global conservation status: None (species is not listed on Natureserve nor has it been assigned a Canadian national conservation status rank). Canadian population size and trends: No information on population size is available. Threats: Residential and commercial development – low (areas where the species occur in Canada are not heavily populated); Agriculture and aquaculture – unknown; Human intrusions and disturbance – medium; Natural system modifications – high (alteration of the natural areas currently protected would likely extirpate the species from Canada); Invasive and other problematic species and genes – unknown but likely medium; Climate change and severe weather – likely to be high (Fernandez-Triana 2014). Small extent of occurrence or area of occupancy: Recorded from two localities in Canada. Limiting biological factors: Unknown.

#### Notes

Materials: see Fernandez-Triana 2014.

### Paroplitis beringianus

Mason, 1981

#### Materials

- **Type status:** Paratype. **Occurrence:** recordedBy: Jose Fernandez-Triana; individualCount: 1; sex: female; **Location:** country: Canada; stateProvince: British Columbia; verbatimLocality: Liard Hot Springs; verbatimElevation: 450 m; **Event:** eventDate: 9-10.vii.1959; **Record Level:** institutionCode: CNC
- **Type status:** Other material. **Occurrence:** recordedBy: Jose Fernandez-Triana; individualCount: 1; sex: male; **Location:** country: Canada; stateProvince: Yukon Territory; verbatimLocality: Top of the World Highway, km 82; verbatimLatitude: 64°05.411'N; verbatimLongitude: 140°57.048'W; **Event:** eventDate: 19.vii.2006; **Record Level:** institutionCode: CNC

#### Distribution

Figs 19, 20

This species is endemic of Alaska (United States), British Columbia and Yukon (Canada) (Fernandez-Triana et al. 2013, Mason 1981). New data on the distribution of the species and photos were published recently (Fernandez-Triana et al. 2013). The Canadian localities (British Columbia, Liard Hot Springs; Yukon Territory, Top of the World Highway, km 82, specimens deposited in the CNC) comprise 50% of the global range of the species. *Paroplitis beringianus* is the only known species of the genus *Paroplitis* in the New World. Nothing is known about the hosts caterpillars parasitized by this braconid wasp.

#### Conservation

**Assessment using the prioritization criteria developed by COSEWIC.** Existing global conservation status: None (species is not listed on Natureserve nor has it been assigned a Canadian national conservation status rank). Canadian population size and trends: No information on population size is available. Threats: Residential and commercial development – low (areas where the species occur in Canada are not heavily populated); Agriculture and aquaculture – unknown; Human intrusions and disturbance – medium; Natural system modifications – high (alteration of the natural areas currently protected would likely extirpate the species from Canada); Invasive and other problematic species and genes – unknown but likely medium; Climate change and severe weather – unknown, but likely to be high because the species is found in relatively fragile Arctic or sub-Arctic environments. Small extent of occurrence or area of occupancy: Recorded from a few localities in northwestern Canada. Limiting biological factors: Unknown.

### Protomicroplitis calliptera

(Say, 1836)

#### Materials

- **Type status:** Other material. **Occurrence:** recordedBy: Jose Fernandez-Triana; individualCount: 1; sex: female; **Location:** country: Canada; stateProvince: Ontario; verbatimLocality: Stitsville; **Event:** eventDate: 22.viii.1963; **Record Level:** institutionCode: CNC
- **Type status:** Other material. **Occurrence:** recordedBy: Jose Fernandez-Triana; individualCount: 1; sex: male; **Location:** country: Canada; stateProvince: Ontario; verbatimLocality: Stitsville; **Event:** eventDate: 30.vi.1963; **Record Level:** institutionCode: CNC
- **Type status:** Other material. **Occurrence:** recordedBy: Jose Fernandez-Triana; individualCount: 1; sex: male; **Location:** country: Canada; stateProvince: Ontario; verbatimLocality: Stitsville; **Event:** eventDate: 10.ix.1963; **Record Level:** institutionCode: CNC

#### Distribution

Figs 21, 22

This species is rather widely distributed in central and eastern United States (Yu et al. 2012), with only a few specimens from Canada being recently reported by Fernandez-Triana 2010. The Canadian specimens (Ontario, Metcalfe and Stitsville, all specimens deposited in the CNC) comprise less than 5% of the global range of the species and the northernmost limit. It has been reported as a parasitoid of two species of *Condica* (Lepidoptera: Noctuidae) (information summarized in Yu et al. 2012).

#### Conservation

**Assessment using the prioritization criteria developed by COSEWIC.** Existing global conservation status: None (species is not listed on Natureserve nor has it been assigned a Canadian national conservation status rank). Canadian population size and trends: No information on population size is available. Threats: Residential and commercial development – medium to high (the areas where the species occur in Canada are already heavily populated); Agriculture and aquaculture – unknown; Human intrusions and disturbance – medium; Natural system modifications – high (alteration of the areas would likely extirpate the species from Canada); Invasive and other problematic species and genes – unknown but likely low, unless another wasp species parasitizing the same host would be introduced (and then competing for the same host, an scenario not likely to occur); Climate change and severe weather – unknown but likely low (climate change increasing the temperatures would not affect much the presence of this species in Canada, because it is already distributed in warmer areas). Small extent of occurrence or area of occupancy: Recorded from two nearby localities in Canada. Limiting biological factors: Host distribution (limited to southern Ontario) may affect the distribution of the wasp in Canada.

### Pseudapanteles gouleti

Fernández-Triana, 2010

#### Materials

- **Type status:** Other material. **Occurrence:** individualCount: 1; **Location:** country: Canada; stateProvince: Ontario; verbatimLocality: Saint Lawrence Islands National Park, Jones Creek by Mallorytown, County Road 5, Mixed Forest (sugar maple and white birch); verbatimElevation: 117 m; verbatimLatitude: 44.4747; verbatimLongitude: -75.865; **Event:** eventDate: 20.vii.2012; **Record Level:** institutionCode: BIO
- **Type status:** Other material. **Occurrence:** individualCount: 1; sex: female; **Location:** country: Canada; stateProvince: Ontario; verbatimLocality: Guelph, near Starkey Hill Conservation Area; verbatimElevation: 320 m; verbatimLatitude: 43.537; verbatimLongitude: -80.134; **Event:** eventDate: 4.viii.2010; **Record Level:** institutionCode: BIO

#### Distribution

Figs 23, 24

All previously known specimens (a total of 23) of *Pseudapanteles gouleti* had been collected in an area bounded by the Saint Lawrence and Ottawa rivers (44–46°N and 74–75°W, for details on localities and collecting dates, see the original description in Fernandez-Triana 2010). Here two new localities are reported for the first time (Ontario, Guelph, near Starkey Hill Conservation Area, and Saint Lawrence Islands National Park, Jones Creek by Mallorytown, County Road 5, specimens deposited in BIO). With the new data, the known distribution of the species is slightly expanded, but still remains an endemic species from southern Ontario (43–46°N and 74–80°W), Canada comprising 100% of the global range for the species. *Pseudapanteles gouleti* is the northernmost known species of the genus *Pseudapanteles*, and has been reported by Fernandez-Triana 2010 as a parasitoid of *Paraclemensia acerifoliella* (Lepidoptera: Incurvariidae).

#### Conservation

**Assessment using the prioritization criteria developed by COSEWIC.** Existing global conservation status: None (species is not listed on Natureserve nor has it been assigned a Canadian national conservation status rank). Canadian population size and trends: No information on population size is available, but the species has been collected over a span of 60 years between mid July to August (with one record on early September). Threats: Residential and commercial development – medium to high (the areas where the species occur are already heavily populated); Agriculture and aquaculture – unknown; Human intrusions and disturbance – medium; Natural system modifications – high (alteration of the areas would likely extirpate the species from Canada); Invasive and other problematic species and genes – unknown but likely low, unless another wasp species parasitizing the same host would be introduced (and then competing for the same host, an scenario not likely to occur); Climate change and severe weather – unknown. Small extent of occurrence or area of occupancy: Recorded from a few localities in Canada. Limiting biological factors: Host distribution (limited to southeastern Canada) may affect the distribution of the wasp in the country.

### Pseudapanteles sesiae

(Viereck, 1912)

#### Materials

- **Type status:** Other material. **Occurrence:** recordedBy: Jose Fernandez-Triana; individualCount: 1; **Location:** country: Canada; stateProvince: Ontario; verbatimLocality: Niagara Falls; **Event:** eventDate: 22.vi.1964; **Record Level:** institutionCode: CNC
- **Type status:** Other material. **Occurrence:** recordedBy: Jose Fernandez-Triana; individualCount: 1; **Location:** country: Canada; stateProvince: Ontario; verbatimLocality: Niagara Falls; **Event:** eventDate: 15.vii.1964; **Record Level:** institutionCode: CNC

#### Distribution

Figs 25, 26

This species is distributed in southern and eastern United States (Yu et al. 2012). Fernandez-Triana 2010 mentioned the species from Canada (Ontario, Niagara Falls), without giving more details. Here complete information of those records is provided for the first time (two specimens deposited in the CNC). The Canadian specimens comprise less than 20% of the global range for the species and the northernmost limit. It has been reported as a parasitoid of *Synanthedon scitula* (Lepidoptera: Sesiidae) (information summarized in Yu et al. 2012).

#### Conservation

**Assessment using the prioritization criteria developed by COSEWIC.** Existing global conservation status: None (species is not listed on Natureserve nor has it been assigned a Canadian national conservation status rank). Canadian population size and trends: No information on population size is available. Threats: Residential and commercial development – high (the areas where the species occur are already heavily populated); Agriculture and aquaculture – medium; Human intrusions and disturbance – high; Natural system modifications – high (alteration of the areas would likely extirpate the species from Canada); Invasive and other problematic species and genes – unknown but likely low, unless another wasp species parasitizing the same host would be introduced (and then competing for the same host, an scenario not likely to occur); Climate change and severe weather – unknown but likely low (climate change increasing the temperatures would not affect much the presence of this species in Canada, because it is already distributed in warmer areas). Small extent of occurrence or area of occupancy: Recorded from one locality in Canada. Limiting biological factors: Host distribution (limited to southeastern Canada) may affect the distribution of the wasp in the country.

### Venanides xeste

(Mason, 1981)

#### Materials

- **Type status:** Other material. **Occurrence:** recordedBy: Jose Fernandez-Triana; **Location:** country: Canada; stateProvince: Ontario; verbatimLocality: Simcoe; **Event:** eventDate: 28.vi.1939; **Record Level:** institutionCode: CNC
- **Type status:** Other material. **Occurrence:** recordedBy: Jose Fernandez-Triana; **Location:** country: Canada; stateProvince: Ontario; verbatimLocality: Simcoe; **Event:** eventDate: 1.vii.1939; **Record Level:** institutionCode: CNC
- **Type status:** Other material. **Occurrence:** recordedBy: Jose Fernandez-Triana; **Location:** country: Canada; stateProvince: Ontario; verbatimLocality: Vineland; **Event:** eventDate: 2.vii.1938; **Record Level:** institutionCode: CNC
- **Type status:** Other material. **Occurrence:** recordedBy: Jose Fernandez-Triana; **Location:** country: Canada; stateProvince: Ontario; verbatimLocality: Chambers Corner; **Event:** eventDate: 5.vii.1940; **Record Level:** institutionCode: CNC
- **Type status:** Other material. **Occurrence:** recordedBy: Jose Fernandez-Triana; **Location:** country: Canada; stateProvince: Ontario; verbatimLocality: Courtland; **Event:** eventDate: 9.vii.1938; **Record Level:** institutionCode: CNC
- **Type status:** Other material. **Occurrence:** recordedBy: Jose Fernandez-Triana; **Location:** country: Canada; stateProvince: Ontario; verbatimLocality: Rondeau Provincial Park; **Event:** eventDate: 8.vii.1940; **Record Level:** institutionCode: CNC
- **Type status:** Other material. **Occurrence:** recordedBy: Jose Fernandez-Triana; **Location:** country: Canada; stateProvince: Ontario; verbatimLocality: St. Williams; **Event:** eventDate: 8.vii.1940; **Record Level:** institutionCode: CNC
- **Type status:** Other material. **Occurrence:** recordedBy: Jose Fernandez-Triana; **Location:** country: Canada; stateProvince: Ontario; verbatimLocality: Saint Lawrence National Park, Thwartway Island; **Event:** eventDate: 16.viii.1976; **Record Level:** institutionCode: CNC
- **Type status:** Other material. **Occurrence:** recordedBy: Jose Fernandez-Triana; **Location:** country: Canada; stateProvince: Manitoba; verbatimLocality: Delta; **Event:** eventDate: 15.vii.1975; **Record Level:** institutionCode: CNC

#### Distribution

Figs 27, 28

This species is widely distributed in the New World, from Brazil to Canada ([Bibr B557207][Bibr B557109], Mason 1981). Here new localities and collecting dates are provided for 20 Canadian specimens deposited in the CNC (Ontario, Simcoe, Vineland, Chambers Corner, Courtland, Rondeau Provincial Park, St. Williams, Saint Lawrence National Park, Thwartway Island; Manitoba, Delta). Canada comprises less than 20% of the global range for the species and the northernmost limit. It has been reported as a parasitoid of several species in the genera *Chionodes* and *Dichomeris* (Lepidoptera: Gelechiidae) (information summarized in Yu et al. 2012).

#### Conservation

**Assessment using the prioritization criteria developed by COSEWIC.** Existing global conservation status: None (species is not listed on Natureserve nor has it been assigned a Canadian national conservation status rank). Canadian population size and trends: No information on population size is available, but specimens have been collected between June and August (although no specimen has been collected since 1976). Threats: Residential and commercial development – medium to high (some of the areas where the species occur are already heavily populated); Agriculture and aquaculture – unknown; Human intrusions and disturbance – medium; Natural system modifications – high (alteration of the areas would likely extirpate the species from Canada); Invasive and other problematic species and genes – unknown but likely low, unless another wasp species parasitizing the same host would be introduced (and then competing for the same host, an scenario not likely to occur); Climate change and severe weather – unknown but likely low (climate change increasing the temperatures would not affect much the presence of this species in Canada, because it is already distributed in warmer areas). Small extent of occurrence or area of occupancy: None. Limiting biological factors: Unknown.

### Venanus heberti

Fernández-Triana, 2010

#### Materials

- **Type status:** Holotype. **Occurrence:** recordedBy: Jose Fernandez-Triana; individualCount: 1; sex: female; **Location:** country: Canada; stateProvince: Prince Edward Island; verbatimLocality: Blooming Point; verbatimElevation: 6 m; verbatimLatitude: 46°24.486'N; verbatimLongitude: 62°57.062'W; **Event:** eventDate: 23.vii.2008; **Record Level:** institutionCode: CNC
- **Type status:** Paratype. **Occurrence:** recordedBy: Jose Fernandez-Triana; **Location:** country: Canada; stateProvince: Nova Scotia; verbatimLocality: Annapolis Royal; **Event:** eventDate: 7.ix.1945; **Record Level:** institutionCode: CNC
- **Type status:** Paratype. **Occurrence:** recordedBy: Jose Fernandez-Triana; **Location:** country: Canada; stateProvince: Nova Scotia; verbatimLocality: Bridgetown; **Event:** eventDate: 2.ix.2012; **Record Level:** institutionCode: CNC
- **Type status:** Paratype. **Occurrence:** recordedBy: Jose Fernandez-Triana; **Location:** country: Canada; stateProvince: Nova Scotia; verbatimLocality: Sable Island; **Event:** eventDate: 11–15.ix.1967; **Record Level:** institutionCode: CNC
- **Type status:** Paratype. **Occurrence:** recordedBy: Jose Fernandez-Triana; **Location:** country: Canada; stateProvince: Nova Scotia; verbatimLocality: Halifax; **Event:** eventDate: 15.viii.1954; **Record Level:** institutionCode: CNC
- **Type status:** Paratype. **Occurrence:** recordedBy: Jose Fernandez-Triana; **Location:** country: Canada; stateProvince: Quebec; verbatimLocality: Knowlton; **Event:** eventDate: 19.viii.1929; **Record Level:** institutionCode: CNC
- **Type status:** Paratype. **Occurrence:** recordedBy: Jose Fernandez-Triana; **Location:** country: Canada; stateProvince: Quebec; verbatimLocality: Kazabazua; **Event:** eventDate: 19.viii.1933; **Record Level:** institutionCode: CNC

#### Distribution

Figs 29, 30

According to the original description (Fernandez-Triana 2010) this species is widely distributed in eastern Canada (Nova Scotia, Annapolis Royal, Bridgetown, Sable Island, Halifax; Prince Edward Island, Blooming Point; Quebec, Knowlton, Kazabazua; all specimens deposited in the CNC). It is the only Canadian endemic of the genus *Venanus* (Canada comprises 100% of the global range for the species), and it has been reported by Fernandez-Triana 2010 as a parasitoid of *Caloptilia asplenifoliella* (Lepidoptera: Gracillariidae).

#### Conservation

**Assessment using the prioritization criteria developed by COSEWIC.** Existing global conservation status: None (species is not listed on Natureserve nor has it been assigned a Canadian national conservation status rank). Canadian population size and trends: No information on population size is available, although the species has been repeatedly collected over a span of 75 years, from mid August to early September. Threats: Residential and commercial development – medium to high (some of the areas where the species occur in Canada are already heavily populated); Agriculture and aquaculture – unknown; Human intrusions and disturbance – medium; Natural system modifications – high (alteration of the natural areas currently protected would likely extirpate the species from Canada); Invasive and other problematic species and genes – unknown but likely low, unless another wasp species parasitizing the same host would be introduced (and then competing for the same host, an scenario not likely to occur); Climate change and severe weather – unknown. Small extent of occurrence or area of occupancy: Recorded from a few localities in eastern Canada. Limiting biological factors: Unknown.

### Venanus pinicola

Mason, 1981

#### Materials

- **Type status:** Holotype. **Occurrence:** recordedBy: Jose Fernandez-Triana; individualCount: 1; sex: female; **Location:** country: Canada; stateProvince: Alberta; verbatimLocality: Banff National Park, Mount Eisenhower; **Event:** eventDate: 19.vii.1958; **Record Level:** institutionCode: CNC
- **Type status:** Paratype. **Occurrence:** recordedBy: Jose Fernandez-Triana; **Location:** country: Canada; stateProvince: Alberta; verbatimLocality: Johnston Canyon; verbatimElevation: 1400 m; **Event:** eventDate: 18.vii.1962; **Record Level:** institutionCode: CNC
- **Type status:** Paratype. **Occurrence:** recordedBy: Jose Fernandez-Triana; **Location:** country: Canada; stateProvince: British Columbia; verbatimLocality: Langford; **Event:** eventDate: 2.viii.1963; **Record Level:** institutionCode: CNC
- **Type status:** Paratype. **Occurrence:** recordedBy: Jose Fernandez-Triana; **Location:** country: Canada; stateProvince: British Columbia; verbatimLocality: Robson; **Event:** eventDate: 21.vii.1949; **Record Level:** institutionCode: CNC
- **Type status:** Paratype. **Occurrence:** recordedBy: Jose Fernandez-Triana; **Location:** country: Canada; stateProvince: British Columbia; verbatimLocality: Hixon; **Event:** eventDate: 1-5.viii.1965; **Record Level:** institutionCode: CNC
- **Type status:** Paratype. **Occurrence:** recordedBy: Jose Fernandez-Triana; **Location:** country: Canada; stateProvince: British Columbia; verbatimLocality: Victoria; **Event:** eventDate: 8.vii.1952; **Record Level:** institutionCode: CNC
- **Type status:** Other material. **Occurrence:** recordedBy: Jose Fernandez-Triana; individualCount: 1; **Location:** country: Canada; stateProvince: Yukon Territory; verbatimLocality: Pelly Crossing; verbatimElevation: 495 m; verbatimLatitude: 62°49.534'N; verbatimLongitude: 136°35.069W; **Event:** eventDate: 15.vii.2006; **Record Level:** institutionCode: CNC

#### Distribution

Figs 31, 32

This species was described by Mason 1981 and considered to be widely distributed in the Nearctic. However, a recent revision of the species combining molecular, biological and geographical data (Fernandez-Triana 2010) found that the species is restricted to western North America. The specimens from eastern Canada mentioned in Yu et al. 2012 actually represent *Venanus heberti* (see above for details on that species). Here complete details on the localities where Canadian specimens of *Venanus pinicola* were collected are reported for the first time (Alberta, Banff National Park, Mount Eisenhower, Johnston Canyon; British Columbia, Langford, Robson, Hixon, Victoria; Yukon Territory, Pelly Crossing; all specimens deposited in the CNC). *Venanus pinicola* has been reported by Fernandez-Triana 2010 as a parasitoid of *Coleotechnites milleri* and *Coleotechnites starki* (Lepidoptera: Gelechiidae).

#### Conservation

**Assessment using the prioritization criteria developed by COSEWIC.** Existing global conservation status: None (species is not listed on Natureserve nor has it been assigned a Canadian national conservation status rank). Canadian population size and trends: No information on population size is available, although the species has been repeatedly collected over a span of 50 years, between July and August. Threats: Residential and commercial development – medium to high (some of the areas where the species occur in Canada are already heavily populated); Agriculture and aquaculture – unknown; Human intrusions and disturbance – medium; Natural system modifications – high (alteration of the natural areas currently protected would likely extirpate the species from Canada); Invasive and other problematic species and genes – unknown but likely low, unless another wasp species parasitizing the same host would be introduced (and then competing for the same host, an scenario not likely to occur); Climate change and severe weather – unknown but likely low (climate change increasing the temperatures would not affect much the presence of this species in Canada, because it is already distributed in warmer areas). Small extent of occurrence or area of occupancy: Recorded from a few localities in western Canada. Limiting biological factors: Unknown.

## Discussion

This paper is the first to consider braconid parasitoid wasps in conservations efforts in Canada. Out of the 28 genera of the subfamily Microgastrinae (Hymenoptera: Braconidae) present in the country, 13 genera and close to 80 species were studied, and 16 species were identified as potential candidates to be included in the Species Candidate Lists of COSEWIC. As such it is just a preliminary effort, to be expanded with more studies in the near future. Based on the analyses made, some recommendations and comments are provided below.

- Because of the relationship that any microgastrine wasp has with its lepidopteran host(s), it will be important to link future conservation efforts with studies done within Lepidoptera. This is an opportunity to work together on the biological and ecological sides of both host and parasitoid species. For example, search for parasitoid wasps can be conducted by rearing caterpillars (e.g. Janzen et al. 2009).
- Several of the species of microgastrine wasps dealt with in this paper are found in protected areas. Having those species added to COSEWIC lists will also increase the profile of those particular protected areas.
- A significant number of the species were collected in or nearby Ottawa. This is a collecting artifact (due to the continue presence of entomologists in the Canadian capital for more than 150 years), but it actually provides an opportunity to explore further the population dynamics of some of those species. Two ideal candidates would be *Lathrapanteles heleios* and *Pseudapanteles gouleti*.
- At least one species (*Microgaster deductor*) might be linked to climate change (Fernandez-Triana 2014), although the current information is not enough to conclude on this matter.
- Two of the species mentioned in this paper (*Distatrix carolinae* and *Exix columbica*) are only known from one specimen, in both cases collected 50–60 years ago. Both might be rare species, or be already extinct, but more collecting effort in the type localities will be needed.

## Supplementary Material

XML Treatment for Alphomelon winniewertzae

XML Treatment for Apanteles samarshalli

XML Treatment for Clarkinella canadensis

XML Treatment for Deuterixys pacifica

XML Treatment for Diolcogaster garmani

XML Treatment for Distatrix carolinae

XML Treatment for Exix columbica

XML Treatment for Lathrapanteles heleios

XML Treatment for Microgaster deductor

XML Treatment for Paroplitis beringianus

XML Treatment for Protomicroplitis calliptera

XML Treatment for Pseudapanteles gouleti

XML Treatment for Pseudapanteles sesiae

XML Treatment for Venanides xeste

XML Treatment for Venanus heberti

XML Treatment for Venanus pinicola

## Figures and Tables

**Figure 1.**
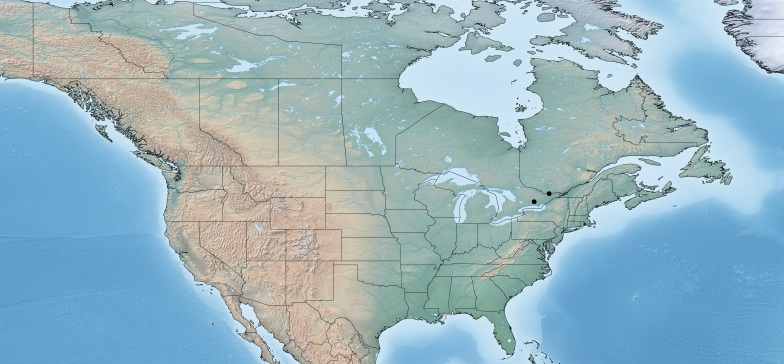
Distribution of *Alphomelon winniewertzae* in Canada.

**Figure 2.**
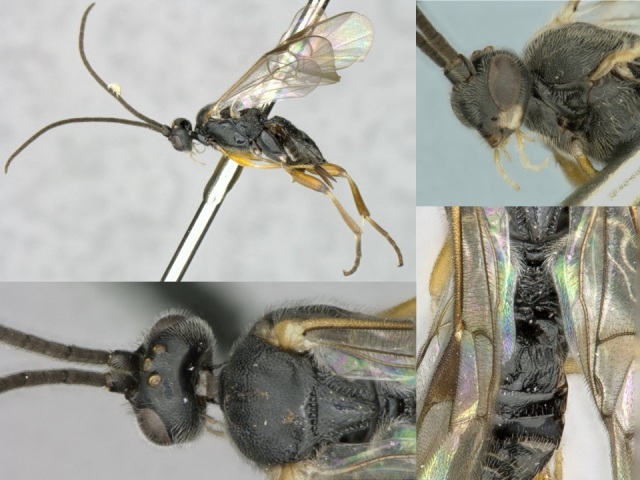
*Alphomelon winniewertzae*, specimen deposited the CNC with code "DNA Voucher CNCHYM 00025".

**Figure 3.**
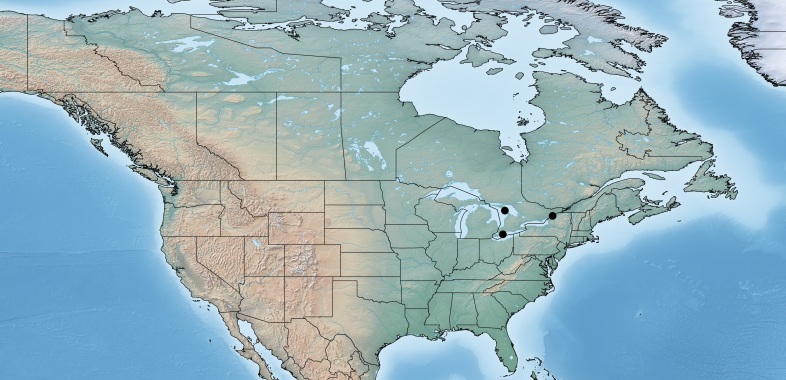
Distribution of *Apanteles samarshalli* in Canada.

**Figure 4.**
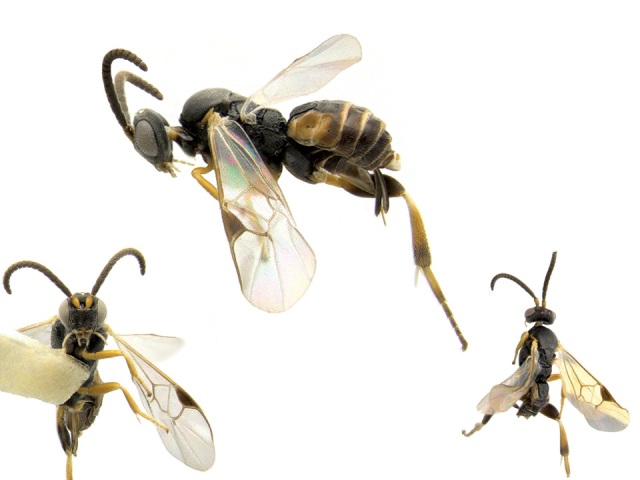
*Apanteles samarshalli*, paratype specimen deposited in the CNC.

**Figure 5.**
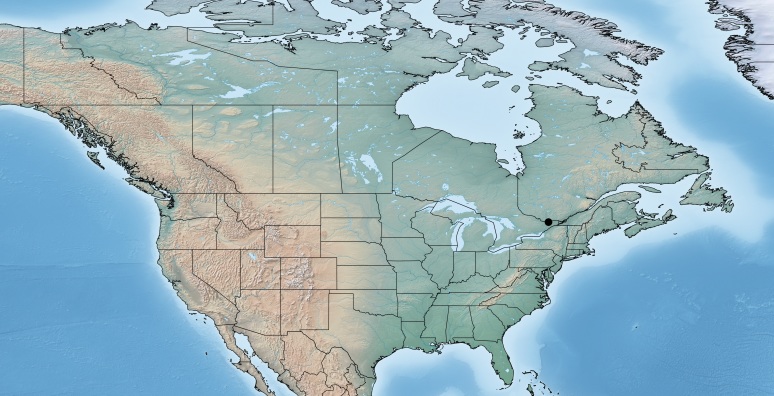
Distribution of *Clarkinella canadensis*.

**Figure 6.**
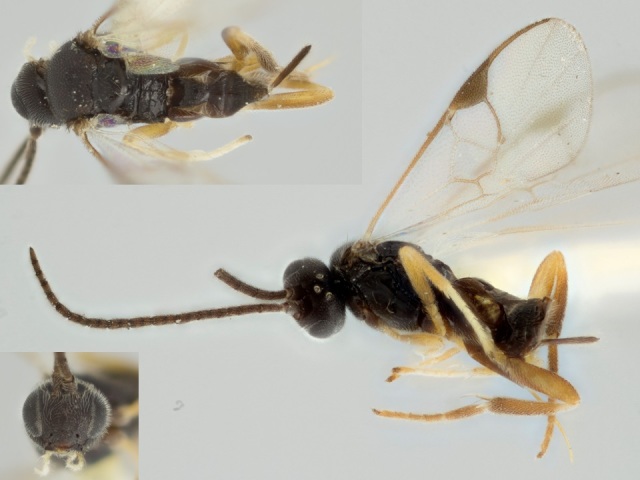
*Clarkinella canadensis*, holotype specimen deposited in the CNC.

**Figure 7.**
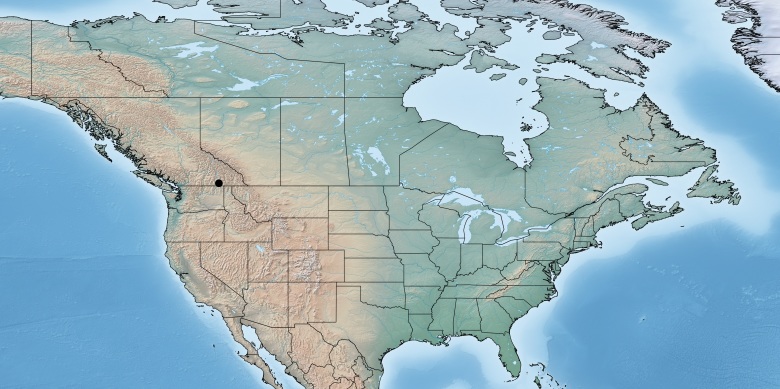
Distribution of *Deuterixys pacifica* in Canada.

**Figure 8.**
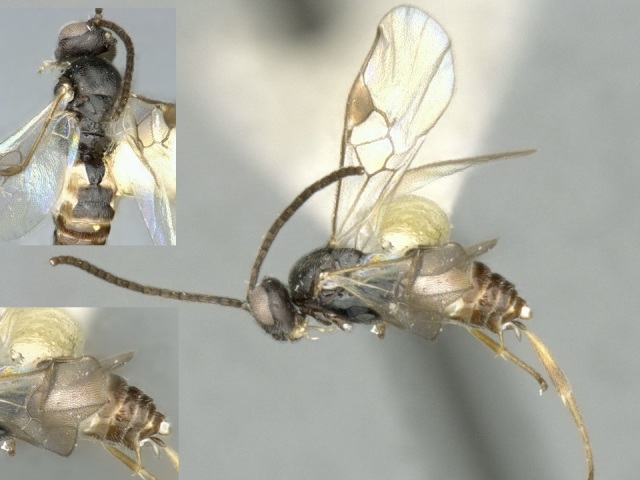
*Deuterixys pacifica*, specimen deposited in the CNC with code "DNA Voucher CNCHYM 00751".

**Figure 9.**
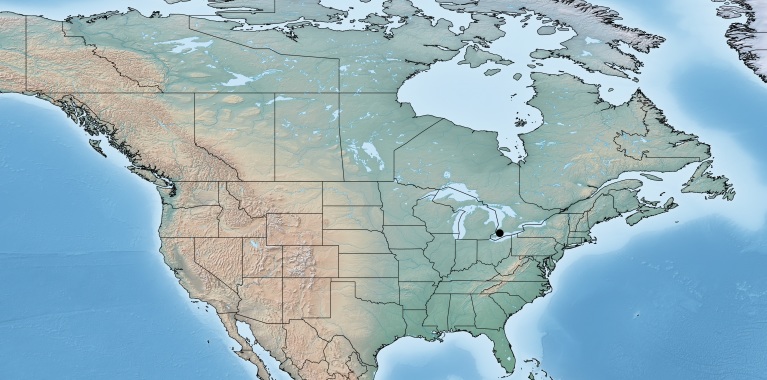
Distribution of *Diolcogaster garmani* in Canada.

**Figure 10.**
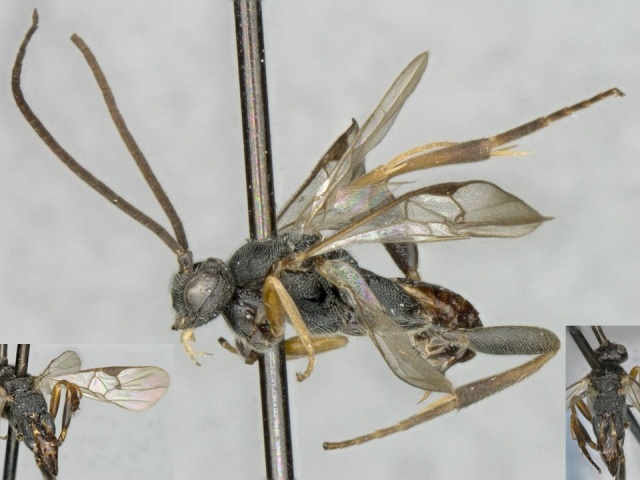
*Diolcogaster garmani*, specimen deposited in the CNC with code "DNA Voucher CNCHYM 00832".

**Figure 11.**
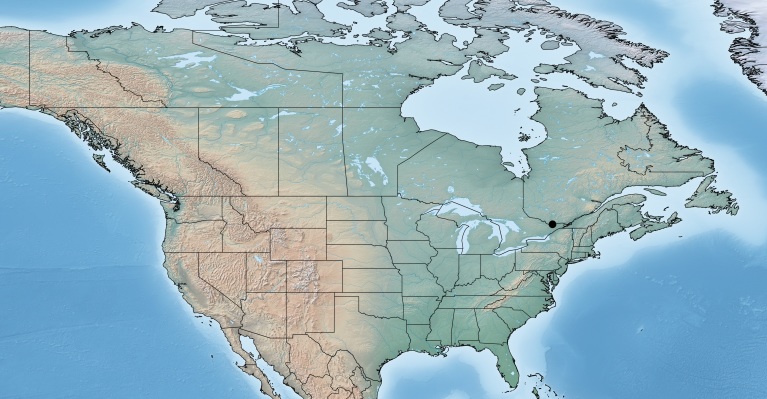
Distribution of *Distatrix carolinae*.

**Figure 12.**
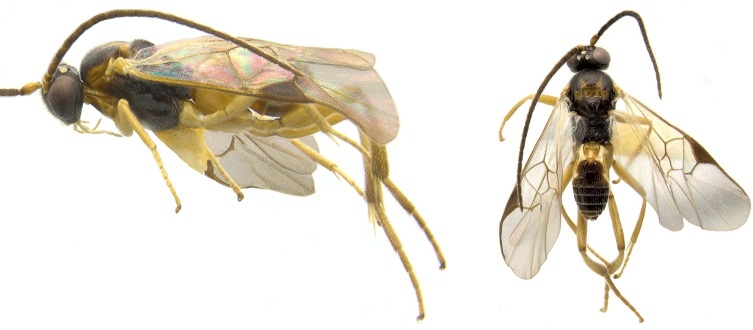
*Distatrix carolinae*, holotype specimen deposited in the CNC.

**Figure 13.**
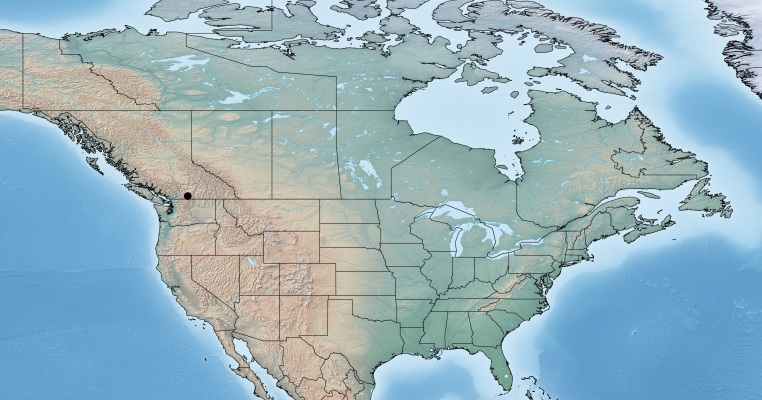
Distribution of *Exix columbica*.

**Figure 14.**
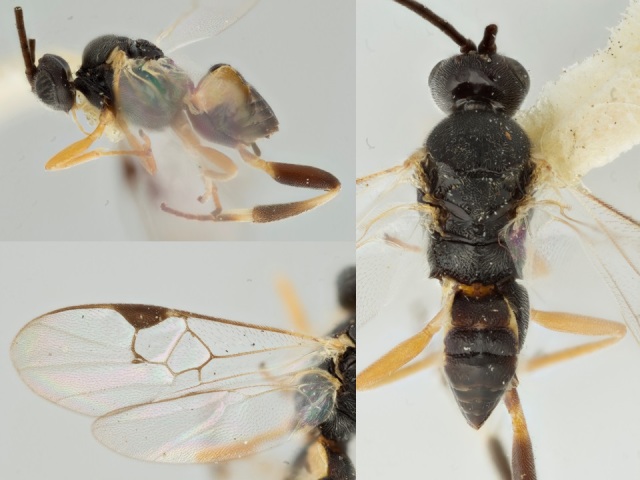
*Exix columbica*, holotype specimen deposited in the CNC.

**Figure 15.**
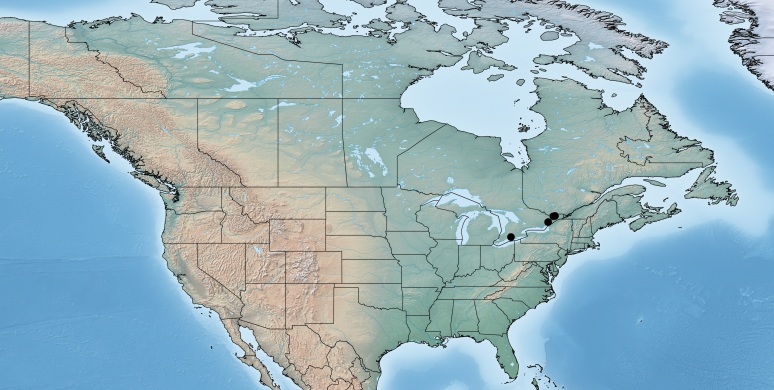
Distribution of *Lathrapanteles heleios*.

**Figure 16.**
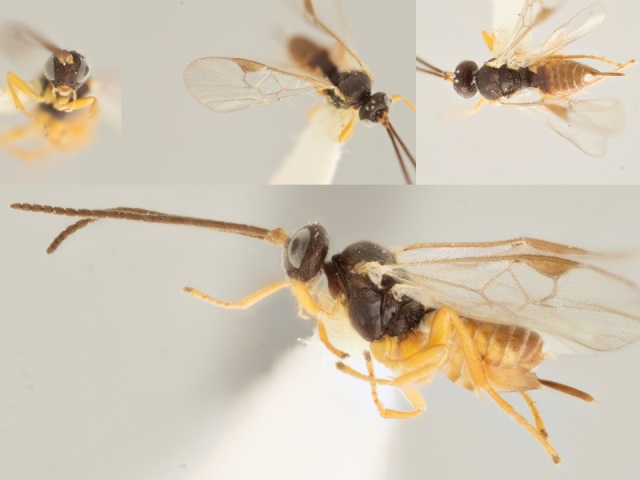
*Lathrapanteles heleios*, holotype specimen deposited in the CNC.

**Figure 17.**
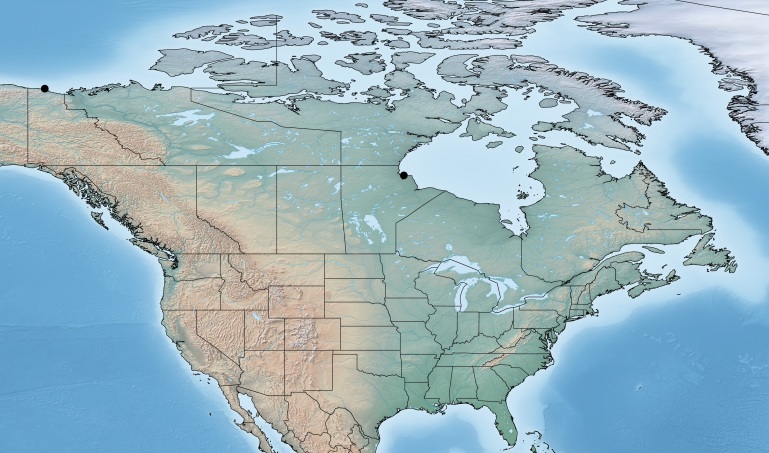
Distribution of *Microgaster deductor* in Canada.

**Figure 18.**
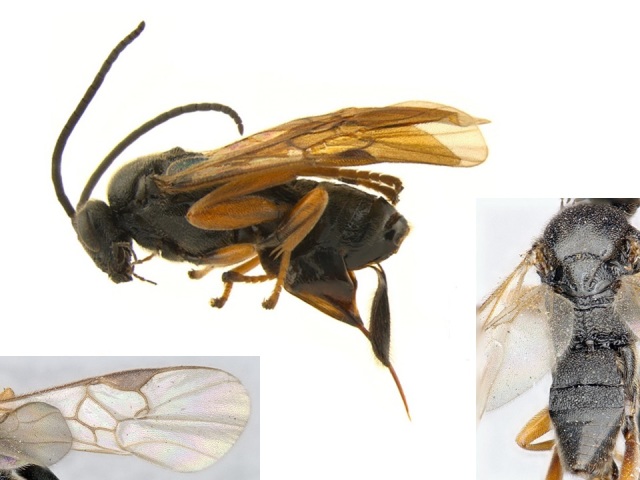
*Microgaster deductor*, photos from two specimens deposited in the CNC.

**Figure 19.**
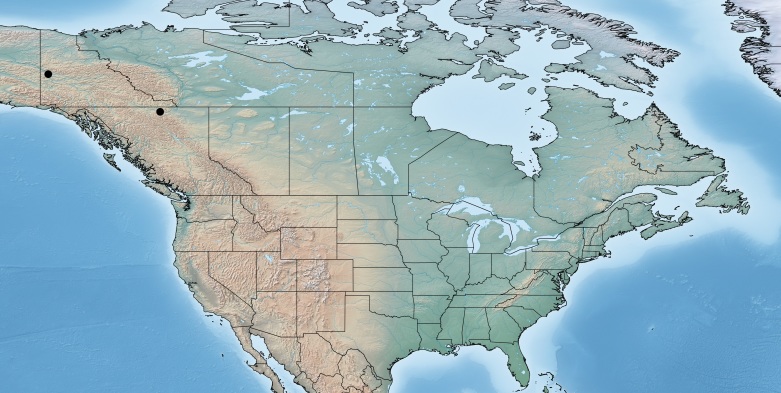
Distribution of *Paroplitis beringianus* in Canada.

**Figure 20.**
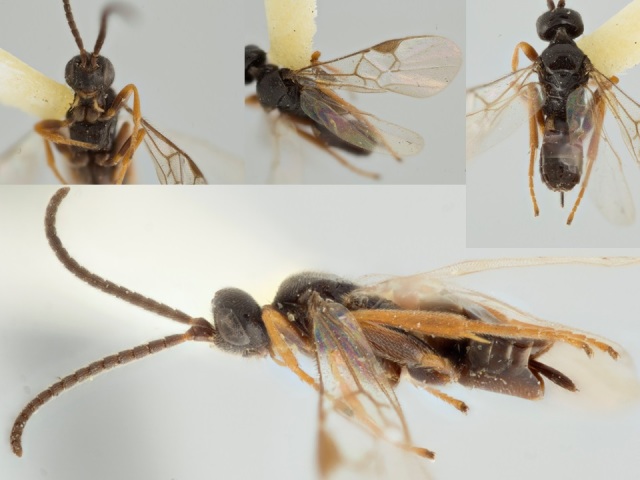
*Paroplitis beringianus*, holotype specimen deposited in the CNC.

**Figure 21.**
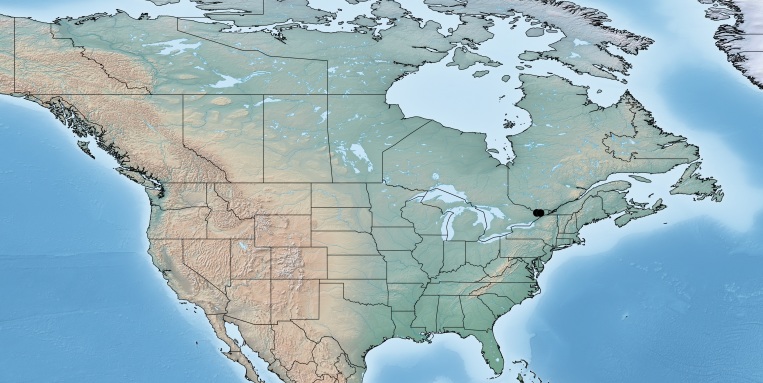
Distribution of *Protomicroplitis calliptera* in Canada.

**Figure 22.**
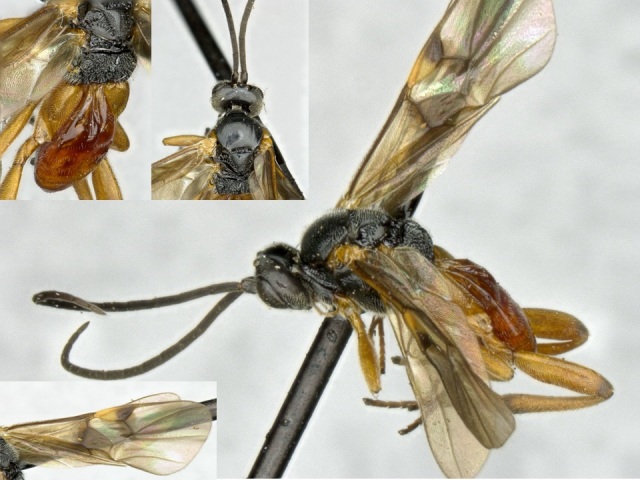
Protomicroplitis calliptera, specimens deposited in the CNC.

**Figure 23.**
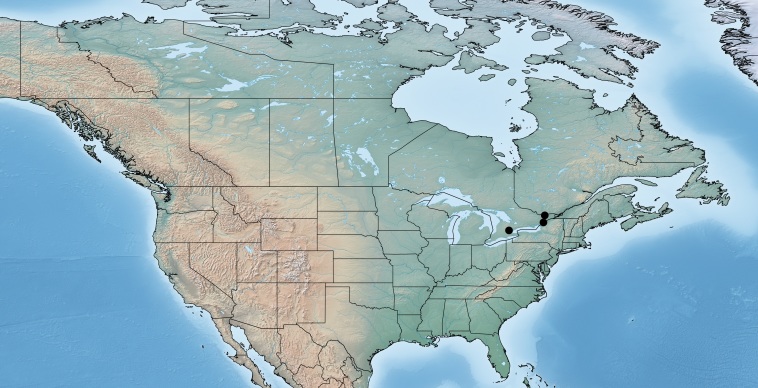
Distribution of *Pseudapanteles gouleti*.

**Figure 24.**
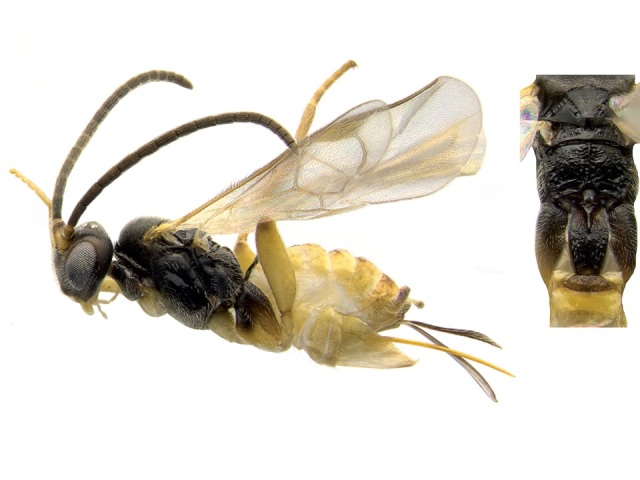
*Pseudapanteles gouleti*, paratype specimen deposited in the CNC.

**Figure 25.**
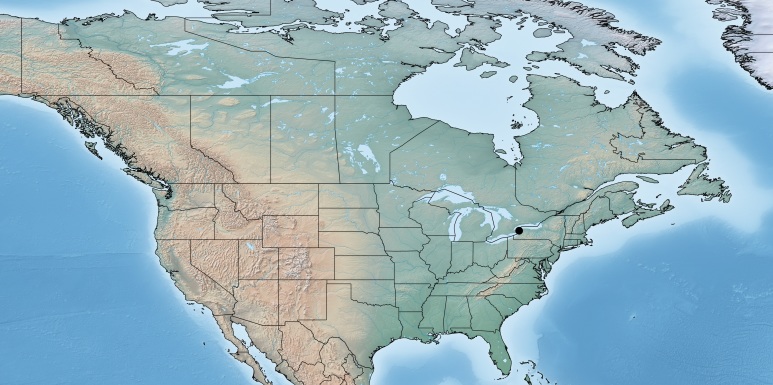
Distribution of *Pseudapanteles sesiae* in Canada.

**Figure 26.**
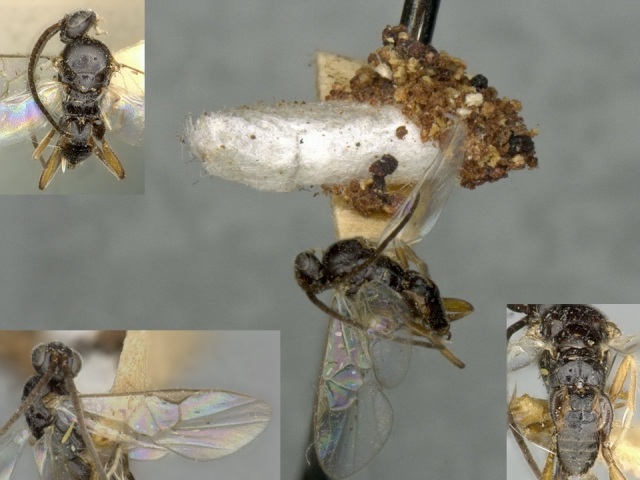
*Pseudapanteles sesiae* specimen deposited in the CNC.

**Figure 27.**
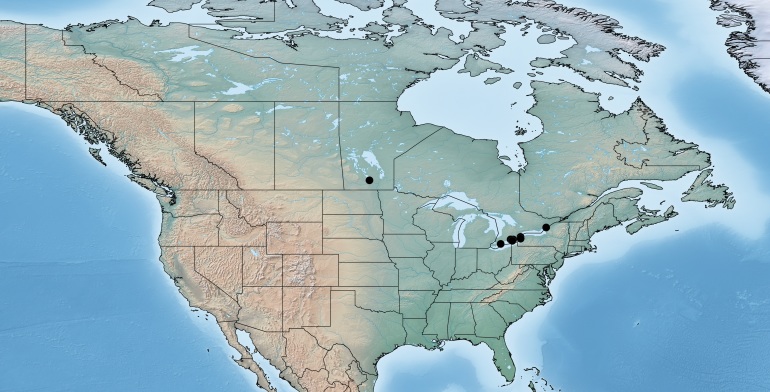
Distribution of *Venanides xeste* in Canada.

**Figure 28.**
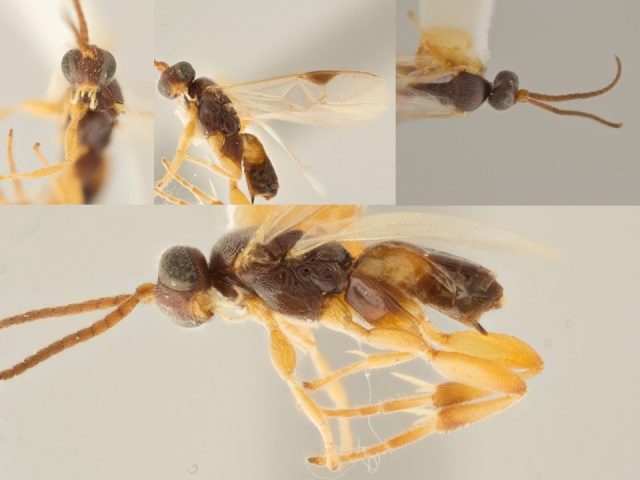
*Venanides xeste*, holotype specimen deposited in the CNC.

**Figure 29.**
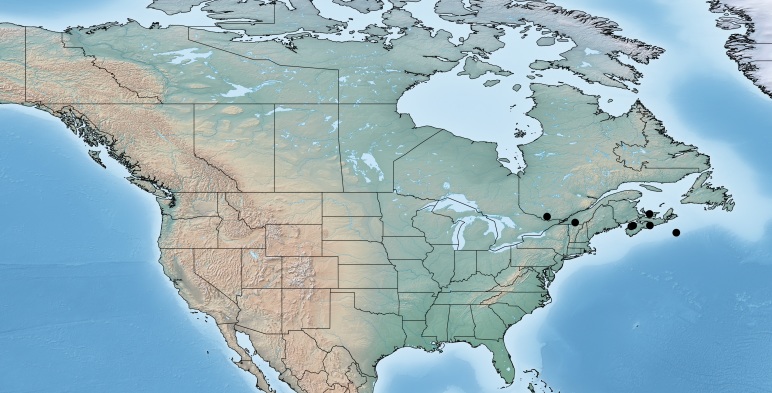
Distribution of *Venanus heberti*.

**Figure 30.**
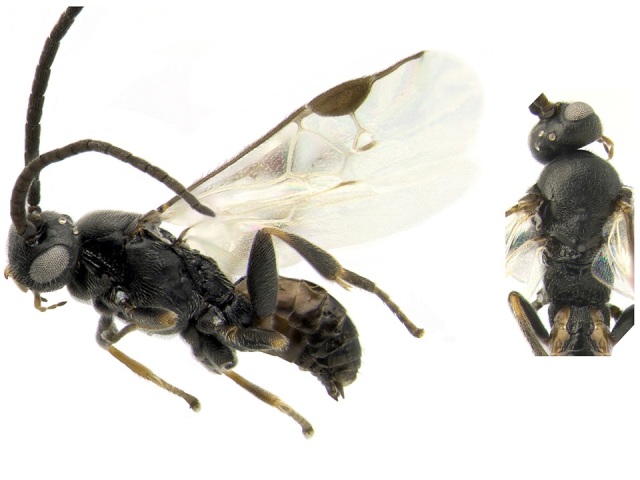
*Venanus heberti*, holotype specimen deposited in the CNC.

**Figure 31.**
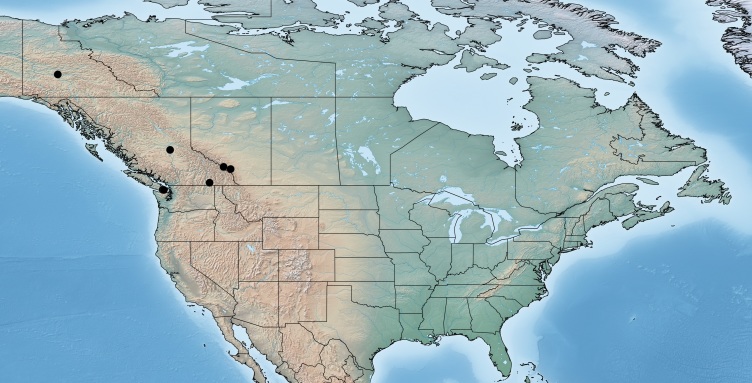
Distribution of *Venanus pinicola* in Canada.

**Figure 32.**
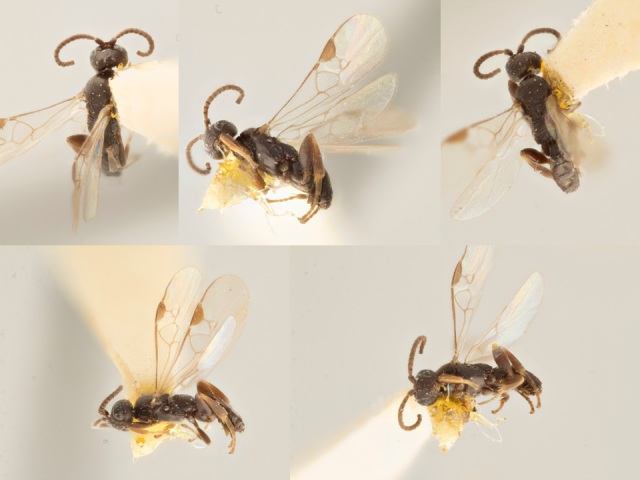
*Venanus pinicola*, holotype specimen deposited in the CNC.
